# Application of a carbon nanoparticle suspension for sentinel lymph node mapping in patients with early breast cancer: a retrospective cohort study

**DOI:** 10.1186/s12957-018-1414-6

**Published:** 2018-06-19

**Authors:** Liulu Zhang, Yijie Huang, Ciqiu Yang, Teng Zhu, Yufeng Lin, Hongfei Gao, Mei Yang, Minyi Cheng, Kun Wang

**Affiliations:** 1 Department of General Surgery, Guangdong General Hospital, Guangdong Academy of Medical Sciences, Guangzhou, 510080 China; 2 Department of Breast Cancer, Cancer Center, Guangdong General Hospital, Guangdong Academy of Medical Sciences, Guangzhou, 510080 China

**Keywords:** Carbon nanoparticle suspension, Breast cancer, Sentinel lymph node biopsy

## Abstract

**Background:**

To stage axillary lymph nodes in women with early-stage breast cancer, sentinel lymph node biopsy (SLNB), rather than axillary lymph node dissection (ALND), has been employed. Moreover, different tracer methods have various advantages and disadvantages. In recent years, carbon nanoparticle suspensions (CNSs) have been used as lymph node tracers during surgeries for thyroid cancer, gastric cancer, and colorectal cancer. The study retrospectively analyzed the feasibility and accuracy of CNS for sentinel lymph node (SLN) mapping in patients with early breast cancer.

**Methods:**

This single-center, retrospective study included breast cancer patients who underwent SLNB from January 1, 2016, to December 31, 2017, in the Department of Breast Cancer, Guangdong General Hospital. All patients received standard SLNB surgery using a CNS tracer.

**Results:**

A total of 332 cases were included in this study. The SLN identification rate was 99.1% (329/332), and the mean number of SLNs was 2.6 (range, 1–6). SLN metastasis was found in 62 (18.8%) cases, of which 90.3% were found to be macrometastases. The sensitivity of SLNB was 95.9% (47/49), with a specificity of 100% (42/42), a positive predictive value of 100% (47/47), a negative predictive value of 95.5% (42/44), and a false-negative rate of 4.1% (2/49).

**Conclusion:**

The identification and predictive values of a CNS tracer for SLNB were satisfactory.

## Background

Axillary lymph node metastasis is one of the most significant prognostic indicators of the long-term prognosis of breast cancer patients [1]. Accurate axillary staging is very important for guiding surgical treatment and choosing the appropriate adjuvant therapy. Sentinel lymph node biopsy (SLNB) has been considered the standard operation for axillary assessment in patients with early-stage breast cancer [2–4]. The traditional method for SLNB relies on the tracing process of either blue dye or radioactive colloid or, in some cases, both [5]. The identification rate of the dual-labeled technology is 89–97%, and the false-negative rate (FNR) is 5–10% [6–11].

Nevertheless, the use of radioactive colloid requires a nuclear medicine department, and there are concerns regarding the exposure of healthcare professionals to radiation [12]. Moreover, complex legislation and restrictions on the disposal and treatment of radioactive substances restrict the widespread use of radioactive colloid. In fact, in China, most patients undergo sentinel lymph node (SLN) mapping with blue dye only because radioisotope is not available at every medical center. However, blue dye-related complications, such as anaphylactic reactions, skin and fat necrosis, local inflammation, and skin staining, have been reported [13–16].

Due to the defects of the traditional SLN tracing method mentioned above, some new techniques have been launched in recent years. A series of studies reported the feasibility of indocyanine green fluorescence (ICG), contrast-enhanced ultrasound with microbubbles, and superparamagnetic iron oxide nanoparticles for SLN mapping in breast cancer [17]. The three methods have clinical potential to challenge the existing standard procedure, but further assessment in randomized trials is needed.

Another promising sentinel lymph node tracer is carbon nanoparticle suspensions (CNSs). With advancements in nanotechnology, CNSs could be widely used in surgeries. CNSs which contain particles with a diameter of 150 nm can pass much more easily through the lymphatic vessels (diameter, 120–500 nm) than through the blood capillaries (diameter, 20–50 nm). Previous studies have demonstrated the safety of CNSs [18]. In recent years, CNSs have been used as lymph node tracers during surgeries for thyroid cancer, gastric cancer, and colorectal cancer [19–21].

The aim of this study is to evaluate the accuracy and feasibility of a CNS for SLN mapping in early breast cancer retrospectively and to provide a clinical alternative technique.

## Methods

### Patients

This single-center, retrospective study included breast cancer patients who underwent SLNB with or without axillary lymph node dissection (ALND) from January 1, 2016, to December 31, 2017, in the Department of Breast Cancer, Guangdong General Hospital. Women with clinical T1-T3N0M0 invasive breast carcinoma were considered eligible. Exclusion criteria included inflammatory breast cancer, former surgical methods in the same axillary region, and stage IV disease. All patients received an ultrasound and a mammography to evaluate axillary lymph nodes prior to surgery. Characteristics suggestive of metastasis are not suitable for SLNB.

### Operative protocol

**Fig. 1:**
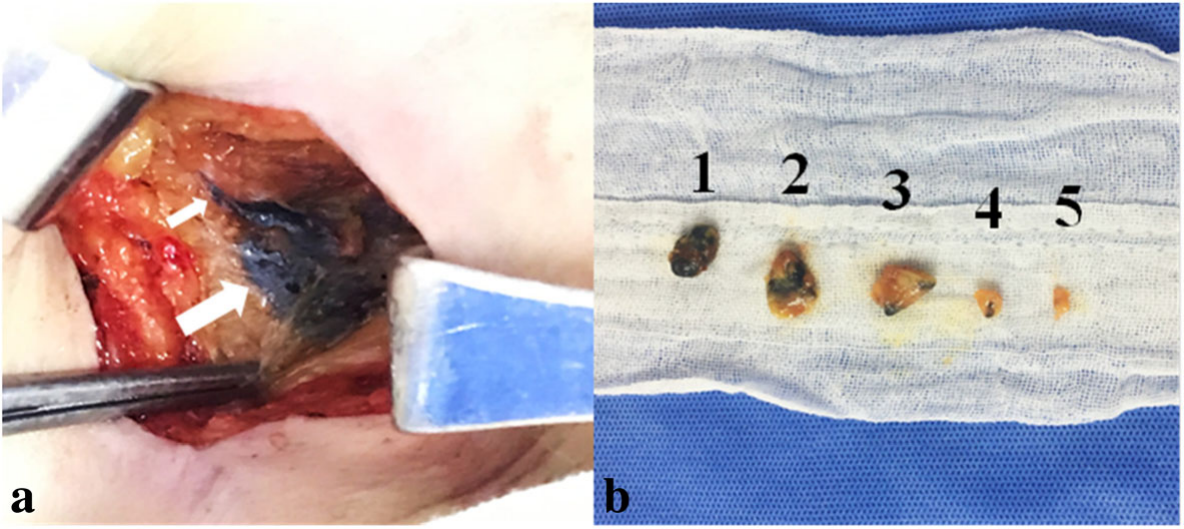
Operative protocol of sentinel lymph node biopsy. **a** A black-stained lymphatic tract (small arrow) leads to a black-stained lymph node (big arrow). **b** Sentinel lymph nodes sorted by dyeing depth

SLNB was carried out using a 1-ml CNS (China Food and Drug Administration approval H20041829, Lai Mei Pharmaceutical Co, Chongqing, China) which was subcutaneously injected into the periareolar area. The breast was massaged for approximately 15 min so that the CNS was absorbed into the lymphatic duct. An incision was made into the skin and subcutaneous tissue, followed by regular separation of the skin flaps. The black-stained lymphatic duct led to the black-stained lymph nodes. After identifying the black-stained lymph nodes, during the exploration period, all black-stained lymph nodes and suspicious lymph nodes were excised (Fig. 1).

### Pathological examination

Intraoperative frozen sectioning and permanent hematoxylin-eosin (H&E) staining were performed on all lymph nodes. Macrometastasis was defined as a tumor with a diameter greater than 2 mm, micrometastasis was defined as tumor deposition from 0.2 to 2.0 mm, and isolated tumor cells (ITCs) were identified as a single cell or a cell cluster of no more than 0.2 mm. A lymph node was deemed positive when a macrometastasis or a micrometastasis was determined.

### Statistical analysis

Data is presented as absolute logarithms and percentages. The sensitivity (Se), specificity (Sp), positive predictive value (PPV), negative predictive value (NPV), and FNR were calculated. SPSS statistical software version 24.0 (SPSS Inc., Chicago, IL, USA) was used to manage all data.

## Results

### Patient characteristics

A total of 332 consecutive patients with primary breast cancer who underwent SLNB mapping with a CNS in Guangdong General Hospital were included in this study. Table 1 shows the patient characteristics. The mean age was 50 years (range, 25 to 77 years). There were 184 (55.4%) patients with T1, 138 (41.6%) with T2, and 10 (3.0%) with T3 tumors (according to the AJCC TNM, 7th edition). Invasive carcinoma of no special type occurred most frequently, accounting for 272 (81.9%) patients. Molecular profiles were classified according to immunohistochemistry (IHC). Ninety (33.3%) patients were classified as luminal A breast cancer, 125 (46.3%) as luminal B breast cancer, 42 (15.6%) as Her-2-positive type breast cancer, and 13 (4.8%) as triple-negative breast cancer. Of these cases, 162 (48.8%) underwent mastectomy, 104 (31.3%) underwent breast-conserving surgery, and 66 (19.9%) underwent nipple-sparing mastectomy (NSM).

**Table 1:** Patient characteristics

Patient characteristics		Number	Percentage
Age (years)	≥ 50	155	46.7
	< 50	177	53.3
Tumor size	T1	184	55.4
	T2	138	41.6
	T3	10	3.0
Pathology	Invasive carcinoma of no special type	272	81.9
	Other	60	18.1
Molecular subtype	Luminal A	90	33.3
	Luminal B	125	46.3
	Her-2-positive	42	15.6
	Triple-negative	13	4.8
Surgery	Mastectomy	162	48.8
	BCS	104	31.3
	NSM	66	19.9

*BCS* breast conserving surgery, *NSM* nipple-sparing mastectomy

### SLN identification

**Fig. 2:**
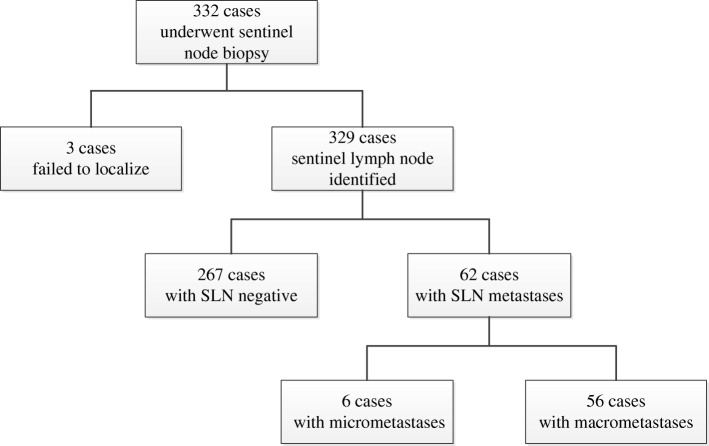
Flow diagram illustrating the results of SLN identification

In 329 of 332 cases, SLNs were identified by CNS. The SLN identification rate was 99.1% (329/332). There were 62 (18.8%) cases with SLN metastases and 56 (90.3%) macrometastases (Fig. 2). The mean number of SLNs identified was 2.6 (range, 1–6).

### Diagnostic value

In total, 91 cases with ALND were included in the results of diagnostic value. In subsequent lymph node dissection, the average number of non-sentinel lymph nodes was 17.7. The pathological examination of axillary lymph nodes revealed that 42 cases were negative and 49 were positive. Forty-seven from the 49 positive cases were detected by SLNB. There were two false-negative cases that the black-stained SLNs were negative, but metastatic lymph nodes (one patient had one and another patient had three) were found in subsequent ALND. Table 2 shows the diagnostic SLNB values. Based on pathological results (the gold standard), the Se was 95.9% (47/49), the Sp was 100% (42/42), the PPV was 100% (47/47), the NPV was 95.5% (42/44), and the FNR was 4.1% (2/49).

**Table 2:** Diagnostic values of sentinel nodes using carbon nanoparticles (*n* = 91)

		ALND		Total	
		Positive	Negative		
SLNB status	Positive	47	0	47	PPV = 100% (47/47)
	Negative	2	42	44	NPV = 95.5% (42/44)
	Total	49	42	91	
		Se = 95.9% (47/49)	Sp = 100% (42/42)	FNR = 4.1% (2/49)	

### Complications of CNS use

**Fig. 3:**
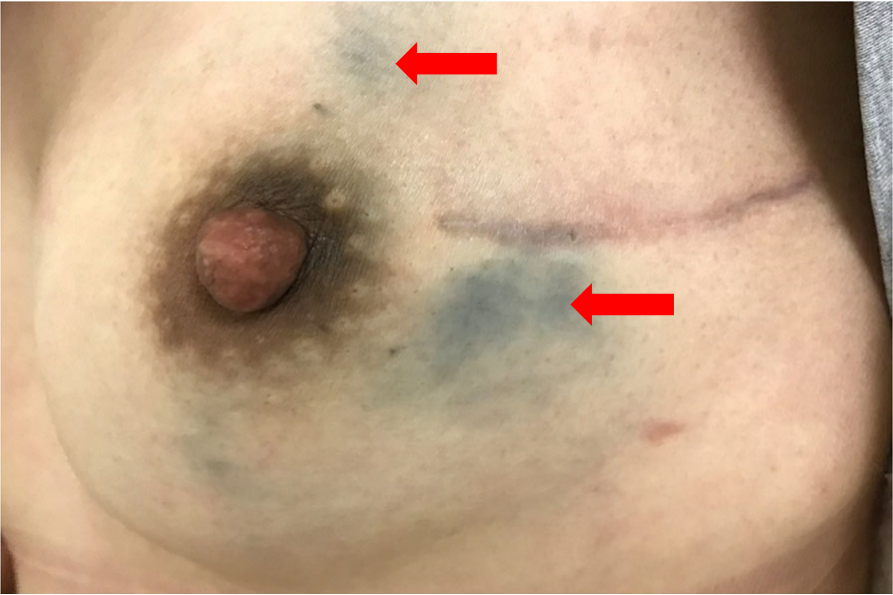
Skin staining at the site of injection

During and after surgery, no patient showed an anaphylactic reaction or a local inflammatory response. No patient experienced skin and fat necrosis. During the median follow-up period, which lasted 13.2 months, 139 patients (41.9%) had skin staining (Fig. 3), most of which showed a faint black tinge, while few patients showed dark staining (Table 3).

**Table 3:** CNS-related complications in sentinel node mapping (*n* = 332)

Event	*n* (%)
Anaphylactic reaction	0
Skin and fat necrosis	0
Local inflammation	0
Skin staining	139 (41.9%)

## Discussion

ALND has been replaced by SLNB for early breast cancer staging of axillary lymph nodes. Blue dye, radioisotopes, and the combination are among the current mapping techniques. Because of the high identification rate, the combination of blue dye and radioactive colloid has been regarded as the gold standard of SLNB [9, 22]. Nevertheless, the disadvantages of radioactive colloid mentioned above limit its application. However, published reports have described a high rate of identification of SLNs when blue dye only was used [23].

Some new techniques, including indocyanine green fluorescence (ICG), contrast-enhanced ultrasound using microbubbles, and superparamagnetic iron oxide nanoparticles for SLN mapping, have emerged in recent years. Compared to isotope, ICG has some advantages such as cheaper price, fewer adverse reactions, and real-time fluorescence imaging during surgery. A study including 847 women with clinical node-negative breast cancer showed that identification rate was 97.2% and the sensitivity was 95.7% with ICG to detect SLNs [24]. A meta-analysis study illustrated that superparamagnetic iron oxide nanoparticles were non-inferior to the standard method for SLN detection that the identification rate was as high as 97.1% and the false-negative rate was 8.4% [25].

However, these techniques require intraoperative detection equipment. CNSs are relatively inexpensive, and no special equipment is required. A prospective study confirmed that CNS resulted in a comparable or better identification rate and diagnostic value than blue dye on marking SLNs in breast cancer [26]. CNS particles have a mean diameter of 150 nm, guaranteeing that they can pass through the lymphatic capillaries and ultimately converge in the lymph nodes which are long enough for the SLNs to be recognized during surgery. In recent years, CNSs have been employed as lymph node tracers during surgeries for thyroid cancer, gastric cancer, and colorectal cancer [19–21]. Animal experiments have demonstrated the safety of CNSs [18]. It was confirmed that CNSs can be used for SLNs mapping in patients with early-stage breast cancer, usually with success [26]. It was also determined that the best time for the maximum coloring of SLNs is 10 to 15 min before surgery, and the dose for a CNS of 1 ml proved to be sufficient.

The current study was designed to retrospectively analyze not only the feasibility but also the accuracy of a CNS tracer for the SLNB procedure. In our study, the identification rate was 99.1%, and the detected value was similar to or better than those reported previously [6–11]. There were three patients in whom we separated the nodes, but no black staining was observed, leading us to believe that SLNs were not successfully detected. When axillary dissection was performed, we did not detect any black-stained lymph nodes or positive lymph nodes in these patients.

Until now, the FNR remained the major evaluation criterion for the routine use of the SLNB technique. According to a meta-analysis of 69 trials, the mean FNR was 8.4% in 8059 patients (5.5–16.7%) [9]. When SLNB was guided by CNS tracers in our study, the FNR was 4.1%, which is clinically acceptable. With a mean SLN number of 2.6 nodes in our study, the SLN number is equal to that reported in other studies that recommended using two to three SLNs to minimize the FNR [27, 28].

SLNB is a reliable and minimally invasive procedure. However, local and systemic complications secondary to the use of blue dye have been reported. Several studies have reported a 1 to 3% incidence of allergic and anaphylactic reactions [29]. Others have reported local skin inflammation or necrosis after blue dye injection [30]. Another drawback of blue dye is a persistent subcutaneous blue stain, with approximately 70% of patients exhibiting staining 3 months after and 41% of patients exhibiting staining 1 year after subdermal injection [14]. In our study, no patient had an anaphylactic reaction or a local inflammatory response. No patient experienced skin and fat necrosis. Skin staining seems to be the most common complication of CNS use.

However, there are still limitations in the current research. CNSs as one of the dyeing methods for SLNB, the problem of skin staining, and how to locate the black-stained lymph nodes need to be improved. Prospective studies and randomized controlled trials are required, and long-term follow-up data on the safety is still needed. In the future studies, we will use the CNS combined with another technique, which may improve the accuracy.

## Conclusion

A carbon nanoparticle suspension tracer for SLNB has favorable identification rates and predictive values.

## Abbreviations

ALND: Axillary lymph node dissection
CNSs: Carbon nanoparticle suspensions
FNR: False negative rate
ICG: Indocyanine green
IHC: Immunohistochemistry
ITCs: Isolated tumor cells
NPV: Negative predictive value
NSM: Nipple sparing mastectomy
PPV: Positive predictive value
Se: Sensitivity
SLNB: Sentinel lymph node biopsy
Sp: Specificity
